# Inactivation of *Escherichia coli* enhanced by anaerobic microbial iron reduction

**DOI:** 10.1007/s11356-020-11209-w

**Published:** 2020-10-20

**Authors:** Lavane Kim, Tao Yan, Van Toan Pham

**Affiliations:** 1 Department of Environmental Engineering, College of Environment and Natural Resources, Can Tho University, 3/2 Street, Xuan Khanh Ward, Ninh Kieu District, Can Tho City, Vietnam; 2 Department of Civil and Environmental Engineering, University of Hawaii at Manoa, Honolulu, HI 96822 USA

**Keywords:** Inactivation, *E. coli*, Microbial iron reduction

## Abstract

Microbial iron reduction (MIR) is an important and ubiquitous natural process in the biogeochemical cycling of iron and carbon in anaerobic sedimentary and subsurface environments. The objectives of this study were (1) to determine if the MIR process can enhance the inactivation of *Escherichia coli* cells under anaerobic conditions and (2) to identify potential inactivation mechanisms. Laboratory microcosm experiments showed that the presence of MIR activity significantly enhanced *E. coli* inactivation, and the inactivation rate under the MIR condition was significantly larger than those under other anaerobic redox conditions. Under anoxic condition, higher Fe^2+^concentrations exhibited a linear function to larger *E. coli* inactivation rates, indicating that the production of Fe^2+^by MIR was one of the important roles in *E. coli* inactivation. When *E. coli* cells were amended as the sole electron source to the MIR process, increased Fe^2+^ production was observed, which corresponded to decreasing TOC concentration. Together, the results suggest that MIR enhanced *E. coli* inactivation through the production of Fe^2+^ as metabolic waste, and the inactivation benefited the MIR process as the inactivated cells were used as an electron source, which represents a potential new mechanism for bacterial inter-species competition. This knowledge could further improve our understanding of the fate of fecal bacteria in natural environments where the MIR process is prevalent, and may also be explored for enhanced removal of bacterial pathogens in engineering processes.

**Electronic supplementary material:**

The online version of this article (10.1007/s11356-020-11209-w) contains supplementary material, which is available to authorized users.

## Introduction

Microbial iron reduction (MIR) is an important and ubiquitous natural process in the biogeochemical cycling of iron and the oxidation of organic matter in anaerobic sedimentary and subsurface environments (Lovley 1991). Although non-enzymatic reduction of Fe(III) under anaerobic conditions can occur (Lovley et al. 1991), dissimilatory MIR by iron-reducing bacteria (IRBs), which use Fe(III) as the terminal electron acceptor in respiration, is considered the most important mechanism in converting Fe(III) to Fe(II)_aq_ (Lovley 1991; Weber et al. 2006). During the MIR process, IRBs can oxidize and mineralize a large variety of organic compounds and produce CO_2_ (Lovley et al. 1989; Lovley and Lonergan 1990; Lovley et al. 1992; Lu et al. 2008), which plays a major role in carbon cycling in anaerobic environments (Lovley 1991). Since Fe(III) substrates are usually insoluble, IRBs and mixed MIR communities including IRBs and archaea have evolved various strategies to transfer electrons, either through direct contact (Reguera et al. 2005) or via intermediate electron carriers (Lovley et al. 1996; Newman and Kolter 2000), to solid Fe(III) substrates. Since the initial isolation of *Shewanella* and *Geobacter* species (Lovley and Phillips 1988; Obuekwe et al. 1981), our knowledge about the phylogenetic diversity of IRBs has greatly expanded to include many species across the domain Bacteria (Lonergan et al. 1996; Lovley 2006), further reflecting the ubiquity of the MIR process in the environment.

The metabolic capability of the MIR process has been extensively explored for biotechnology applications, including using Fe(III) to enhance the bioremediation of organic pollutants (Lovley 1991) and using the MIR communities to transfer electrons to external anodes for energy harvesting in microbial fuel cells (Bond et al. 2002). However, the possibility of using the MIR process in water purification, particularly in bacterial pathogen inactivation and removal, has not been investigated. Although anaerobic environments in general favor bacterial pathogen survival, the MIR process contains several features that may enhance pathogen inactivation. First of all, amorphous ferric oxides are known to be strong adsorbent for bacterial cells (Kapetas et al. 2012; Mills et al. 1994), which could concentrate bacterial cells on surfaces where MIR occurs. Second, the MIR process produces Fe^2+^ as a metabolic waste, which was recently shown to be a powerful bactericidal agent under anoxic conditions (Auffan et al. 2008; Kim et al. 2010; Lee et al. 2008). Thirdly, the metabolic diversity of IRBs and mixed MIR communities in degrading a large variety of organic substrates (Lovley et al. 1989; Lovley and Lonergan 1990; Lovley et al. 1992; Lu et al. 2008) indicates that cellular materials of inactivated fecal bacteria cells may be used as carbon and energy sources, which is supported by *Shewanella*’s capability of consuming extracellular DNA (Gödeke et al. 2011).

Therefore, the objectives of this study were (1) to determine if MIR process can enhance the inactivation of the model organism *Escherichia coli* under anaerobic conditions and (2) to identify potential inactivation mechanisms. We hypothesis that survival of *E. coli* in sediment would be more strongly affected by MIR process than other processes under anoxic condition. Laboratory microcosms were established to compare the inactivation of *E. coli* cells in the presence/absence of MIR activity and between the MIR condition and other anaerobic redox conditions. *E. coli* inactivation in the presence of different Fe^2+^ concentrations was quantified to verify the bactericidal effect of Fe^2+^ under anoxic condition. The capability of the MIR process to use *E. coli* cells as the sole electron source for energy metabolism was also investigated.

## Materials and methods

### Bacterial strains, cultivation, and enumeration

*E. coli* ATCC 29522 was used as the model fecal bacterium in this study. A fresh single colony from overnight growth on a LB agar plate was used to inoculate LB broth, which was cultivated at 37°C with constant shaking at 200 rpm. Stationary-phase cells (OD_600_ > 1.2) were collected by centrifugation at 10,000×*g* for 3 min. The cell pellet was washed by resuspending in 0.1× PBS buffer (pH 7.2) and then pelleting by centrifugation for five times to remove residual broth nutrients. The cell pellet was subsequently resuspended in a sterile artificial freshwater medium (see below for composition) to prepare cell stock solution with a target OD_600_ of 0.8, which contained approximately 10^9^–10^10^ CFU mL^−1^, and was used in subsequent inactivation experiments. *E. coli* in the stock solution and in samples collected from the experiments was enumerated by spread plating of appropriate 10-fold sequential dilutions on the mTEC agar (USEPA 2002).

### Iron-coated sand preparation

Quartz sand was coated by amorphous ferric oxyhydroxide (FeOOH) following the procedure described by (Mills et al. 1994). Sand was heated at 550°C for 3 h and then rinsed several times with DI water to remove organic matters. Trace metal was washed by soaking the sand in concentrated 10 mol L^−1^ HCl for 24 h, then rinsing in 0.01 mol L^−1^ NaOH, and finally rinsed with DI water until the effluent pH reached 8.0 ± 0.1. Sand was dried at 110 °C and then stored in a clean bottle for later use. The cleaned dry quartz sand was immersed in 400 mL of FeCl_3_ solution (50 g L^−1^ of FeCl_3_.H_2_O, pH 1.9), and 30 mL of NaOH (0.5 mol L^−1^) was added instantaneously followed by gradual addition of 1 mL NaOH (0.5 mol L^−1^) until pH reaches 4.5–5.0. The mixture was then shaken for 36 h to allow further coating of FeOOH onto sand surfaces. Iron-coated sand was then rinsed with DI water, air dried, and saved in a clean bottle for later use.

### Microbial inoculum preparation

Anaerobic microbial inocula were first enriched using FeOOH, SO_4_^2−^, and NO_3_^−^ as the terminal electron acceptor for iron-reducing bacteria (IRBs), sulfate-reducing bacteria (SRBs), and denitrifiers, respectively. Artificial freshwater medium used in the enrichment contained 2.5 g L^−1^ NaHCO_3_, 0.1 g L^−1^ NaCl, 0.1 g L^−1^ KCl, 0.1 g L^−1^ MgCl_2_.6H_2_O, 0.1 g L^−1^ CaCl_2_.2H_2_O, 1.5 g L^−1^ NH_4_Cl, 0.6 g L^−1^ NaH_2_PO_4_, 0.005 g L^−1^ MnCl_2_.4H_2_O, 0.001 g L^−1^ NaMoO_4_, and 0.05 g L^−1^ yeast extract (Lovley and Phillips 1988). The enrichments were established in 160-mL serum bottles, containing 100 mL of artificial freshwater medium and 10 mmol L^−1^ of sodium acetate as the electron donor. The microcosm for IRB enrichment contained 20 g of FeOOH-coated quartz sand, the microcosm for SRB enrichment contained 10 mmol L^−1^ of MgSO_4_, and the microcosm for denitrifiers contained 10 mmol L^−1^ MgNO_3_. The microcosms were inoculated with 1 g of anaerobic sediment sample collected from a pond near the Waipahu Stream (21° 23′ 05.9″ N, 158° 00′ 46.4″ W) where iron-rich Haplustoll soil is present. The serum bottles were capped with rubber stopper and sealed with aluminum crimp caps. Air in the head space of serum bottles was removed by vacuum and followed by 15 min N_2_ bubbling for three times. The microcosm was incubated at room temperature in dark, and the enrichment of IRBs, SRBs, and denitrifiers was verified by Fe^2+^ accumulation, SO_4_^2−^ concentration reduction, and NO_3_^−^ concentration reduction, respectively (data not shown). To pool the three inocula into one anaerobic inoculum, the microcosms were thoroughly shaken by hand for 3 min, and 10 mL of each suspension was withdrawn with a syringe and injected into an anoxic serum bottle. The microbial community of the pooled anaerobic inoculum was determined by Illumina sequencing of 16S rRNA gene following the procedure described by Zhang et al. (2015). The microbial community contained common IRBs, SRBs, and denitrifiers (Table S1).

### Microcosm setup with different redox conditions

Anaerobic microcosms to determine the decay kinetics of *E. coli* cells under different redox conditions were established in the same way as the inoculum enrichment described above. Four sets of microcosms, each containing three independent microcosms as biological replicates, were established. Two sets of identical microcosms contained FeOOH-coated quartz sand, with one set receiving the anaerobic inoculum (termed active MIR microcosms) and the other set receiving no inoculum and hence remaining sterile (control microcosms). The active MIR microcosms and the control microcosms were used to compare the effect of active MIR activity on *E. coli* inactivation. The microcosms receiving sulfate were to establish microbial sulfate reduction (MSR) condition, while the microcosms receiving nitrate were to establish microbial nitrate reduction condition (MNR). The MSR and MNR microcosms were used to compare with the *E. coli* inactivation in the MIR microcosms. The pooled anaerobic inoculum (1 mL) was injected into the MIR, MSR, and MNR microcosms using a syringe. Equal amounts of *E. coli* cells (ca. 3 × 10^9^ CFU) were also injected into all microcosms using freshly prepared *E. coli* stock solutions. The microcosms were then incubated at room temperature in dark without shaking. Samples were collected daily by first vigorously shaking serum bottles for 2 min followed by immediately withdrawing 1 mL of the mixtures using a syringe.

### Inactivation of *E. coli* by Fe^2+^

The impact of different Fe^2+^ concentrations on the inactivation of *E. coli* was investigated in 50 mL serum bottles under anoxic conditions. Each bottle contained 30 mL of 0.1× PBS buffer that was deoxygenated by flushing N_2_ into the headspace for 15 min before sealed off with a rubber stopper and aluminum seal. *E. coli* cells from the stock solution were injected into the bottles to reach an initial cell concentration of approximately 10^7^CFU mL^−1^. Different final concentrations of Fe^2+^ (0, 0.05, 0.1, 0.2, and 0.5 mmol L^−1^) were added into the bottles to make the experimental treatments. Each treatment used three bottles as biological replicates. The bottles were incubated under the anoxic condition at room temperature (22 ± 0.5°C) on a shaker at 40 rpm. Samples were taken from each bottle (1 mL) after fully mixing at different incubation times (0, 1, 12, 36, and 60 h). The samples were subjected to 10-fold serial dilution in sterile 0.1× PBS buffer, and the culturable *E. coli* cells were enumerated as described above.

### Carbon source experiment

Three sets of MIR microcosms, each in triplicate, were also established to test if *E. coli* cells could be used as the sole carbon source for MIR. The basic microcosms setup was the same as described above, except for the carbon source. The microcosms either received sodium acetate (final concentration 0.2 mmol L^−1^), *E. coli* cells (ca. 10^10^ CFU), or no carbon source (control). The carbon source equivalency of the spiked *E. coli* cells was determined by first autoclaving the samples at 121 °C for 20 min to lyse the cells and then analyzing the cell lysates by TOC analysis (described below), which gave an average TOC of 232.6 mg C L^−1^. The acetate concentration used (0.2 mmol L^−1^) gives a theoretical TOC of 4.8 mg C L^−1^, and the measured TOC concentration was 2.7 ± 0.3 mg C L^−1^. The microcosms were inoculated by injecting 1 mL of the anaerobic inoculum using a syringe. All microcosms were incubated at room temperature, in dark, and without shaking. The microcosms were sampled daily using the same procedure described above, and the samples were analyzed for Fe^2+^ and TOC.

### Chemical analysis

Fe^2+^was measured using the colorimetric ferrozine assay (Stookey 1970) following the procedure described by Lovley and Phillips (1986). Briefly, liquid samples (0.1 mL) were transferred into 5 mL of ferrozine (1 g L^−1^) in 50 mmol L^−1^ HEPES (N-2-hydroxyethylpiperazine-N′-2-ethanesulfonic acid) buffer (pH 7). The mixtures were vortexed for 15 s and then filtered through a Nuclepore filter (0.2 μm). Filtrates were measured for light absorbance at wavelength 562 nm using a spectrometer (Hach DR/4000 U), and Fe^2+^ concentration was calculated based on standard curves established by using ferrous ethylene-diammonium sulfate as standard solutions. Anions (SO_4_^2−^ and NO_3_^−^) were quantified by using a Dionex ICS-1100 Ion Chromatograph equipped with a 4 mm AS14A analytical column. 25 μL of samples was injected into the analytical system by an AS-DV auto-sampler utilizing Dionex filter cap vials that automatically filter the samples before loading into injection loop. TOC concentration in the samples was determined by using a TOC analyzer (Shimadzu).

### Data analysis

The Fe^2+^ concentration difference between the sterile control microcosms and active MIR microcosms was tested using *t* test for individual sampling dates. The Fe^2+^ production rate during the three sequential periods following repetitive *E. coli* cell spikes were determined by linear regression of Fe^2+^ concentration data versus time. *E. coli* cell inactivation was modeled using the 1st-order model ($\mathrm{Ln}\kern0.1em \left(\frac{C_t}{C_0}\right)=-{k}_{\mathrm{d}}t\kern-0.25em$), where C_t_ and *C*_0_ are the concentrations of *E. coli* cells in the microcosms at time *t* and time zero, respectively. *k*_d_ is the decay coefficient of the 1st-order decay model and was identified through linear regression of natural log-transformed concentration data. A comparison of *E. coli* inactivation rates was performed using ANCOVA with Tukey’s post hoc test. ANOVA was used to test if there was difference in *E. coli* concentration in the Fe^2+^ inactivation experiment, and to test if Fe^2+^ and TOC concentrations were different among the different carbon source treatments. Statistical tests were conducted either in the Microsoft Excel with a statistiXL plug-in or using Sigma 10.0, and the default significant level is 0.05, unless stated otherwise.

## Results

### *E. coli* inactivation in the presence of MIR

**Fig. 1:**
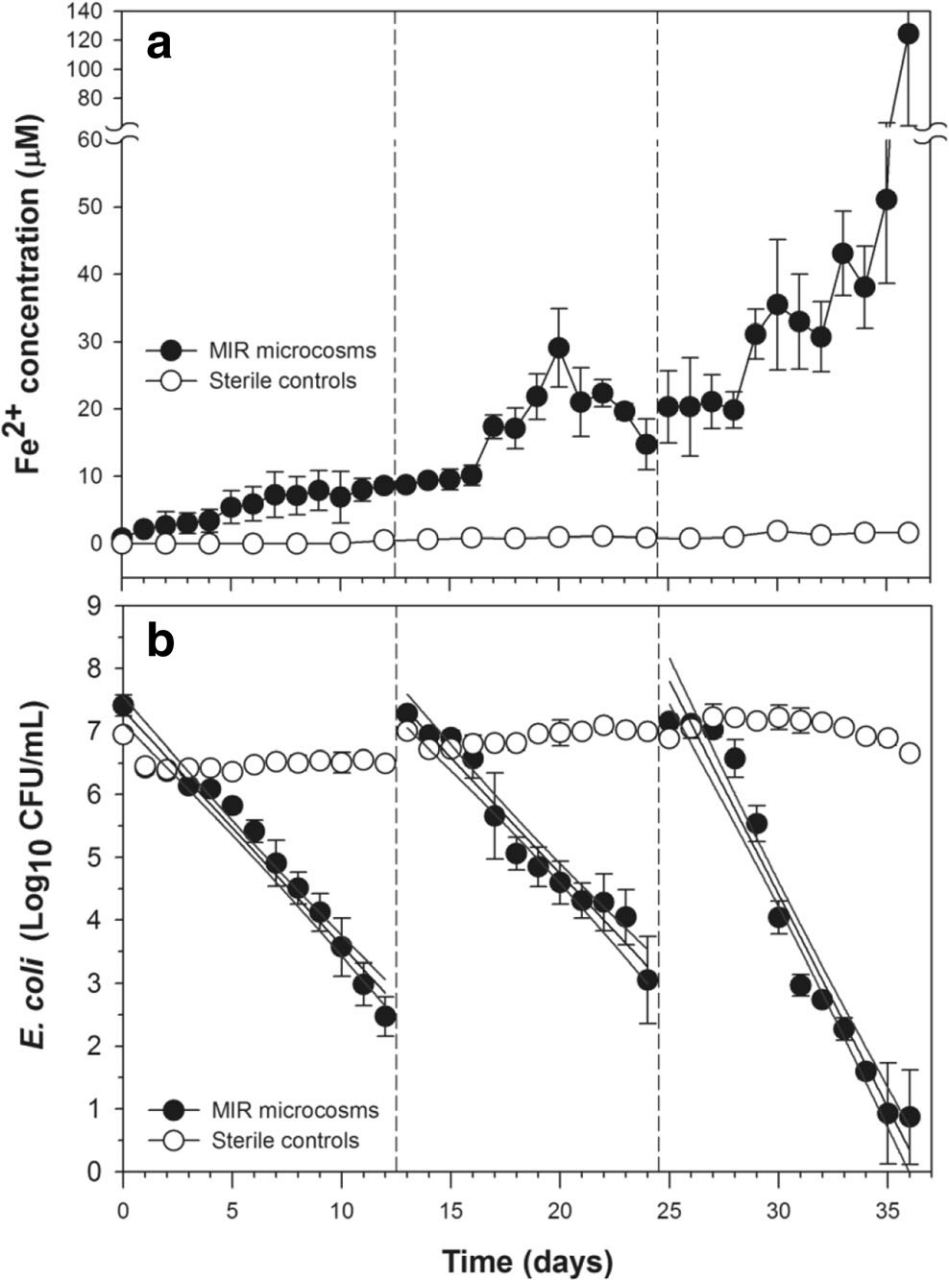
Production of Fe^2+^ (**a**) and inactivation of *E. coli* cells (**b**) in the active microbial iron reduction and control microcosms. Repetitive spike of *E. coli* cells (c.a. 10^7^ CFU mL^−1^) occurred on days 0, 12, and 24, as indicated by the dashed lines. Error bars indicate the standard deviation of the mean of triplicate microcosms

The impact of MIR on the inactivation of *E. coli* cells was investigated by comparing the decay patterns of *E. coli* cells in the active MIR microcosms, which received the anaerobic inoculum, to those in the control microcosms, which did not receive the anaerobic inoculum. During the incubation, Fe^2+^ concentration in the active MIR microcosms continued to increase over time, and became significantly higher than that in the control microcosms after day 1 (*t* test, *P* < 0.05), indicating successful development of MIR activity (Fig. 1a). The Fe^2+^ production rates were calculated to be 0.65, 1.20, and 5.78 μmol L^−1^ day^−1^ for the three periods following the three repetitive spikes of *E. coli* cells on days 0, 13, and 25, respectively. The Fe^2+^ production rate in the second period was higher than that in the first period (although not statistically significant, *P* = 0.829); the Fe^2+^ production rate was significantly higher in the third period than in the first two periods (ANCOVA, *P* < 0.001), indicating a continuous and significant increase in MIR activity over time.

Significantly faster inactivation of *E. coli* cells was observed in the active MIR microcosms than in the control microcosms following all three repetitive spikes of *E. coli* cells (Fig. 1b). The control microcosms exhibited negligible *E. coli* inactivation during the experimental course. In the active MIR microcosms, *E. coli* exhibited first-order inactivation rates of 0.86, 0.85, and 1.56 day^−1^in the three periods following the three repetitive spikes, respectively (Table 1). A significant difference was detected among the three decay rates, and Tukey’s post hoc tests showed that there was no significant difference in the inactivation rates during the first two periods (0.86 and 0.85 day^−1^); inactivation rate after the third spike (1.56 day^−1^) was significantly higher than those observed after the first two periods (ANCOVA, *P* < 0.001). The increasingly higher *E. coli* inactivation rates corresponded well to the increasingly higher MIR activity over the experimental course.

**Table 1:** Average inactivation rates of *E. coli* cells in microcosms under different redox conditions (*k*_MIR_, *k*_MNR_, and *k*_MSR_), goodness-of-fit of the linear regression (*r*^*2*^), and rate comparisons*

Spike	*k*_MIR_	*r*^2^	*k*_MNR_	*r*^2^	*k*_MSR_	*r*^2^	*k*_MIR_ > *k*_MNR_	*k*_MIR_ > *k*_MSR_
1	0.86 ± 0.03	0.95	0.74 ± 0.04	0.88	0.24 ± 0.02	0.78	*P* = 0.04	*P* < 0.001
2	0.85 ± 0.05	0.91	0.45 ± 0.05	0.73	0.27 ± 0.03	0.65	*P* < 0.001	*P* < 0.001
3	1.56 ± 0.06	0.95	0.87 ± 0.06	0.84	0.60 ± 0.04	0.89	*P* < 0.001	*P* < 0.001

*ANCOVA with Tukey’s post hoc test

### Comparison with other anaerobic redox conditions

**Fig. 2:**
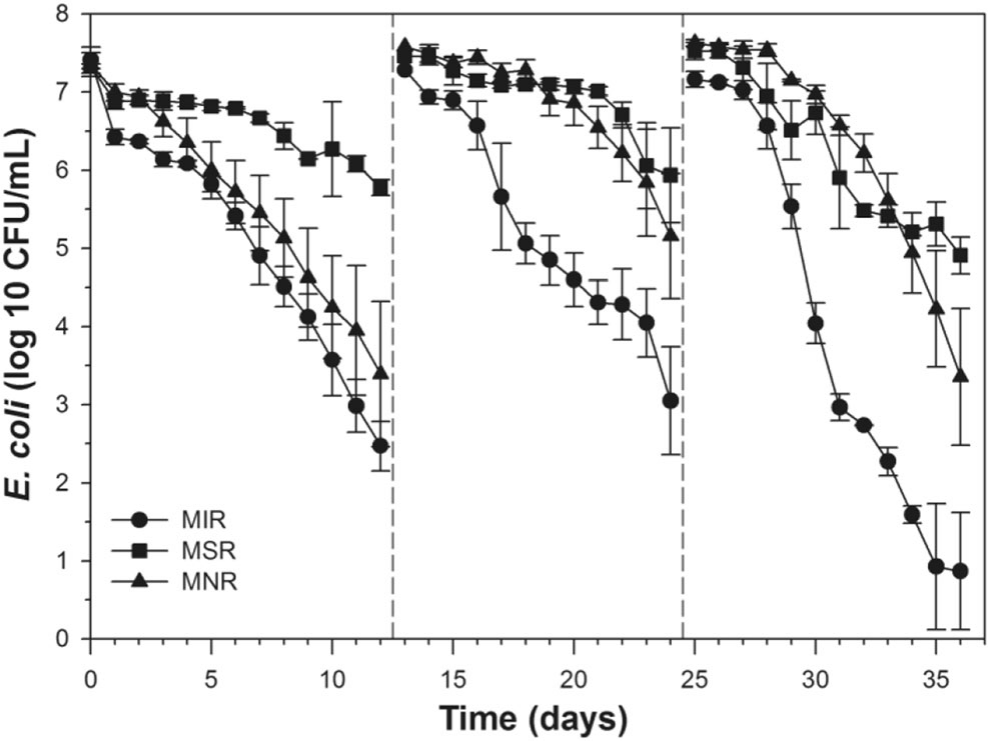
Inactivation of *E. coli* cells under different redox conditions (microbial iron reduction, microbial sulfate reduction, and microbial nitrate reduction). Repetitive spike of *E. coli* cells (c.a. 10^7^ CFU mL^−1^) occurred on days 0, 12, and 24, as indicated by the dashed lines. Error bars indicate the standard deviation of the mean of triplicate microcosms

To further test the impact of MIR activity on *E. coli* inactivation, the MIR microcosms were also compared with the MSR and MNR microcosms, which had the same microcosm setup as the MIR microcosms, receiving the same anaerobic inoculum, but were provided with SO_4_^2−^ or NO_3_^−^, respectively, to establish different anaerobic redox conditions. Microbial sulfate reduction and nitrate reduction were developed in the MSR and MNR microcosms, respectively, as indicated by the gradual decrease of SO_4_^2−^ and NO_3_^−^ concentration over time (Figure S1). The different microcosms exhibited different *E. coli* inactivation patterns, with the MIR microcosms showing the fastest inactivation after all three repetitive spikes (Fig. 2). Fitting of the *E. coli* inactivation data to the first-order model gave inactivation rates of 0.45–0.87 day^−1^ and 0.24–0.60 day^−1^ for the MNR and MSR microcosms, respectively, which were significantly smaller than the those in the MIR microcosms after all three repetitive spikes of *E. coli* cells (ANCOVA, *P* ≤ 0.04) (Table 1).

### Inactivation of *E. coli* cells by ferrous ion

**Fig. 3:**
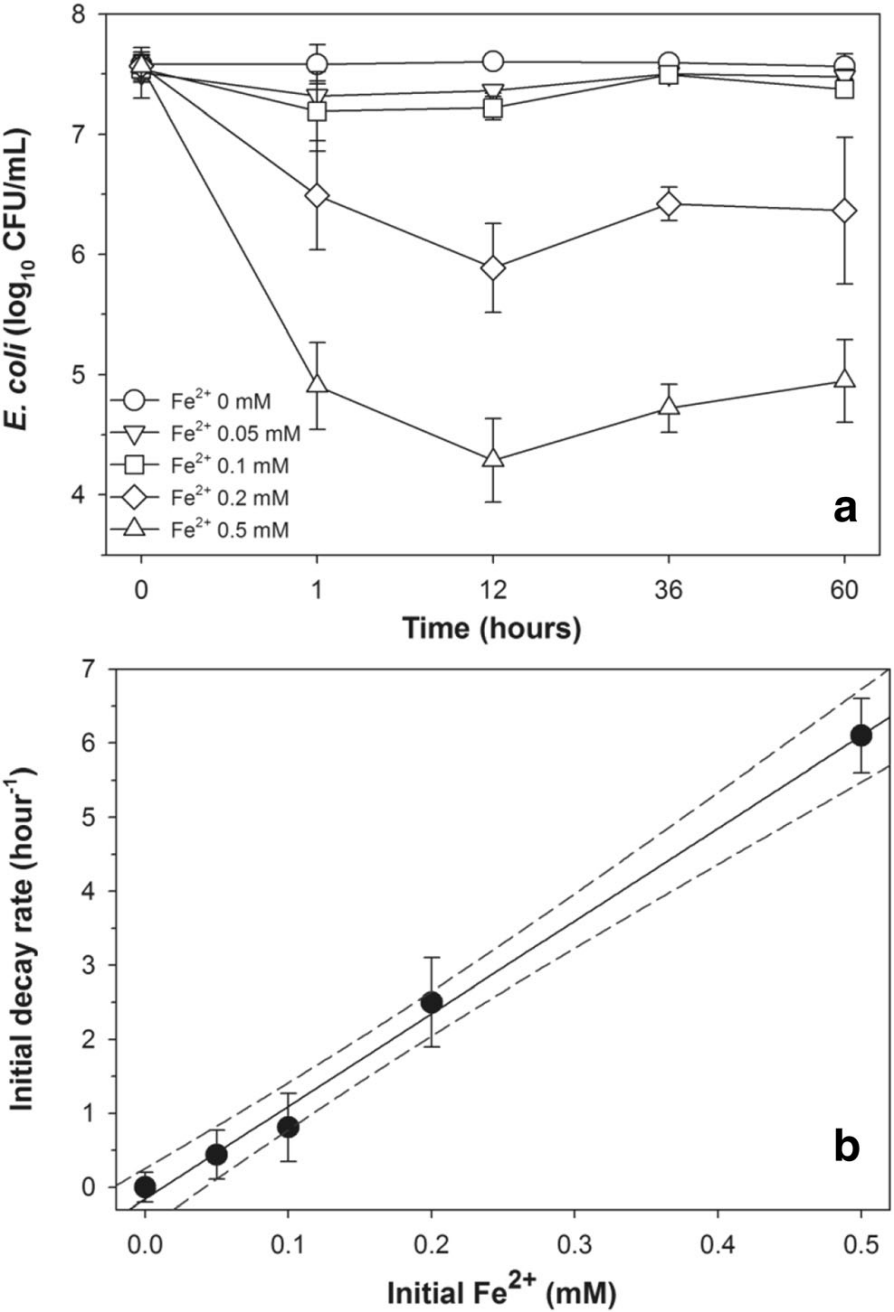
Inactivation of *E. coli* under different Fe^2+^ concentration over different exposure times (**a**), and linear regression between the first-hour inactivation rate and Fe^2+^ concentration (**b**). Error bars indicate the standard deviation of the mean of triplicate microcosms. The dashed lines in compartment B represent 95% confidence bands

The bactericidal effect of Fe^2+^produced from the MIR activity was investigated by determining *E. coli* inactivation rates in the presence of different Fe^2+^ concentrations under anoxic condition (Fig. 3a). *E. coli* inactivation was negligible in the absence of Fe^2+^over the entire 60 h of incubation, while significantly lower *E. coli* concentrations were observed in the presence of Fe^2+^within the first hour of incubation (ANOVA, *P* < 0.01). Since majority of *E. coli* inactivation occurred within the first hour and then tailed off almost completely, the initial *E. coli* inactivation rates were calculated using the inactivation data within the first hour (Fig. 3b). Higher Fe^2+^ concentrations corresponded to larger *E. coli* inactivation rates; the microcosms receiving 0, 0.05, 0.1, 0.2, and 0.5 mmol L^−1^ Fe^2+^ exhibited initial decay rates of 0.001, 0.44, 0.81, 2.5, and 6.1 h^−1^, respectively. The observed *E. coli* inactivation rates showed a linear relationship to Fe^2+^ concentrations, as indicated by the excellent goodness-of-fit (*r*^*2*^ = 0.99).

### *E. coli* cells as sole electron source

**Fig. 4:**
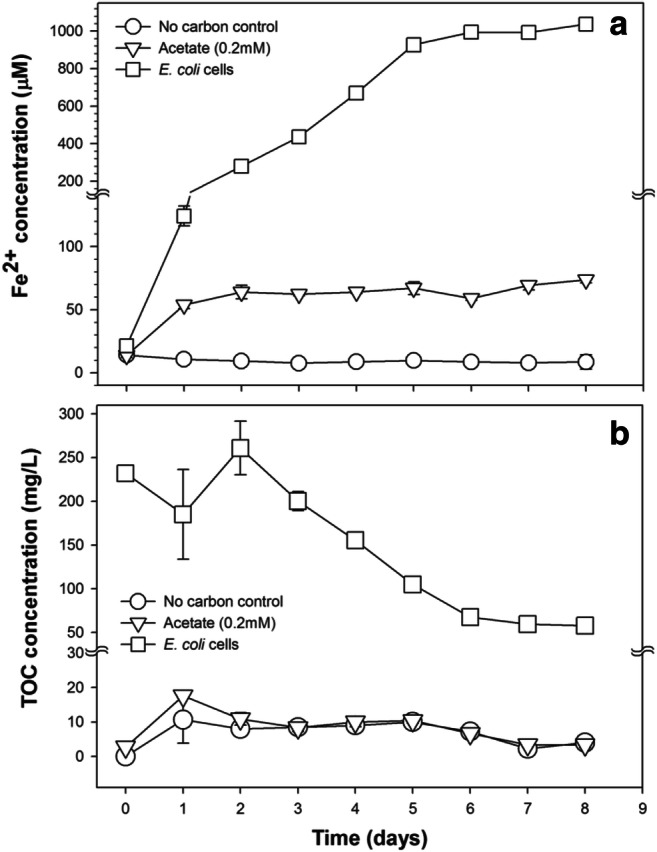
Fe^2+^ concentrations in microbial iron reduction microcosms that received no carbon, acetate, or *E. coli* cells as the sole electron source (**a**), and their corresponding TOC concentration change over time (**b**). Error bars indicate the standard deviation of the mean of triplicate microcosms

The capability of MIR to use *E. coli* cells as the sole electron source was determined by comparing Fe^2+^ production in MIR microcosms that received *E. coli* cells (2.8 × 10^10^ CFU mL^−1^) to two sets of control microcosms that received either 0.2 mmol L^−1^ either acetate (positive control) or no carbon source (negative control) (Fig. 4a). On day 0, there was no significant difference in Fe^2+^ concentration among all microcosms (ANOVA, *P* = 0.99). Starting from day 1 to day 8, the positive control microcosms showed a Fe^2+^ concentration range of 50.6 to 75.7 μmol L^−1^, which were significantly higher than those in the negative controls (ANOVA, *P* < 0.001), indicating the development of active MIR activity in the microcosms. The negative control microcosms showed very low Fe^2+^ concentrations and negligible fluctuation over the eight-day experimental course (concentration range: 2.6–15.4 μmol L^−1^).

Starting from day 1, the microcosms that received *E. coli* cells as the sole carbon source showed significantly higher concentrations of Fe^2+^ than both sets of control microcosms (ANOVA, *P* < 0.001). The Fe^2+^ concentration continued to increase over time, reached 995.2 ± 26.1 μmol L^−1^ on day 6, and then gradually tailed off. Correspondingly, initial TOC concentrations in the MIR microcosms that received *E. coli* cells was also significantly higher than the two sets of control microcosms, and its decreasing pattern over time, which tailed off after day 6, coincided with the Fe^2+^ concentration increase pattern.

## Discussions

The impact of MIR activity on the inactivation of *E. coli* cells was clearly demonstrated by the significantly faster decay in the active MIR microcosms than in the sterile control microcosms, which was repetitively observed over the three sequential spikes of *E. coli* cells (Fig. 1b and Table 1). Since the only difference between the active MIR and the control microcosms was the anaerobic inoculum, the observed difference in decay patterns should be primarily attributed to the presence of the anaerobic inoculum and resulting MIR activity in the active MIR microcosms. The limited *E. coli* concentration reduction over time in the control microcosms indicates that abiotic factors, such as cell recovery from the iron-coated surface (Kapetas et al. 2012; Mills et al. 1994) and abiotic inactivation by iron oxide surface (Asadishad et al. 2013), made a limited contribution to the reduction of culturable *E. coli* cell number under the experimental conditions. Endogenous decay of *E. coli* cells under sterile anaerobic conditions is usually very slow (Feng et al. 2010), which is in line with the slow *E. coli* inactivation observed in the sterile control microcosms.

The correspondence between higher MIR activity and faster inactivation rate (Fig. 1) and the faster inactivation under the MIR condition than under the MNR and MSR redox conditions (Fig. 2) provided further support to the association between MIR activity and enhanced *E. coli* inactivation. In the active MIR microcosm, increasingly higher Fe^2+^ production rates over time indicate continuous growth of iron-reducing bacteria and higher MIR activity in the third period than in the first two (Fig. 1b). This is in agreement with the slow growth of IRBs; for example, the growth rate of *Geobacter metallireducens* was reported to be below 0.003 h^−1^ (Marozava et al. 2014).

Comparing *E. coli* inactivation in the MIR microcosms to that in the MNR and MSR microcosms provided further support to the superiority of MIR on *E. coli* inactivation. The MIR, MNR, and MSR microcosms started with the same anaerobic inoculum, and the development of respective anaerobic activities followed the normal expectation of typical growth rates of the individual anaerobic organisms (i.e., MNR > MSR > MIR). Although during the first spike, there was no significant difference in inactivation rates between the MIR and MNR microcosms, significantly faster inactivation of *E. coli* cells was observed in the MIR microcosms than the other microcosms during both the second and third spikes. The increasingly larger difference in decay rates between the MIR microcosms and other microcosms as time progressed corresponded well to its slower growth rate in comparison to MNR and MSR, suggesting that even faster inactivation could be achievable with higher MIR activity.

The observation of significantly faster *E. coli* inactivation in the presence of MIR activities highlights the importance of biotic stresses to the inactivation of fecal bacteria. Several recent studies have shown that *E. coli* often exhibit significantly faster decay rates in the presence of indigenous microbiota in soil (Bogosian et al. 1996; Medema et al. 1997), freshwater (Bogosian et al. 1996; Medema et al. 1997), seawater (Carlucci et al. 1961), and beach sand (Feng et al. 2010). Different types of biotic stresses, including protozoa predation (Enzinger and Cooper 1976; Gonzalez et al. 1992), phage infection (Ashelford et al. 2003; Rozen and Belkin 2001), and bacterial competition (Feng et al. 2010; Hibbing et al. 2010; Jannasch 1968; Mitchell and Nevo 1965; Mitchell et al. 1967), have been shown to play significant roles in *E. coli* inactivation in the environment. The significant difference in *E. coli* inactivation between the different redox conditions indicates that bacterial competition from the MIR community played a significant role in the observed *E. coli* cell inactivation.

Bacterial interspecies competition can involve many different mechanisms, such as nutrient competition and antibiotic production (Hibbing et al. 2010). Although bacterial metabolic wastes are generally considered to be adverse to the waste producer themselves, their impact to other bacterial populations, particular those allochthonous to the prevailing microbial community, has not been explicitly studied. Previous works have shown that Fe^2+^ can inactivate *E. coli* cells under anoxic conditions (Auffan et al. 2008; Kim et al. 2010; Lee et al. 2008), and results in this study suggest that the Fe^2+^ produced as a metabolic waste of MIR can rapidly inactivate *E. coli* cells (Fig. 3) and hence may function as a mechanism in bacterial interspecies competition. Since many soil bacteria can produce secondary metabolites (antibiotics) to participate in interspecies competition (D’Costa et al. 2006), it is not totally surprising bacterial metabolic wastes may also fulfill similar ecological functions.

Regarding the chemical mechanism underlying the inactivation, previous authors suggested that Fe^2+^ could have reacted with intracellular H_2_O_2_ to produce reactive oxygen species (such as hydroxyl radical) via the Fenton’s reaction (Kim et al. 2010; Lee et al. 2008), which are strong oxidants with bactericidal effects (Imlay 2013). Aerobically grown *E. coli* cells typically generate 14 μmol L^−1^ of H_2_O_2_ per second (Seaver and Imlay 2001) and maintain a steady-state 0.1–0.2 μmol L^−1^ of H_2_O_2_ due to various scavenging mechanisms (Gonzalez-Flecha and Demple 1995). Although the intracellular H_2_O_2_ produced under aerobic condition could have persisted and been carried over into the anoxic condition used in this study, the linear dependency of *E. coli* inactivation on Fe^2+^ concentration observed here and in previous studies (Auffan et al. 2008; Kim et al. 2010; Lee et al. 2008) suggest Fe^2+^, rather than intracellular H_2_O_2_, was the limiting factor, hence partially dissuading the contribution from Fenton’s reaction. Further researches needed to fully elucidate the inactivation mechanism(s).

The inactivated *E. coli* cells were used as the sole electron source in the MIR process, as indicated by the increase in Fe^2+^ concentration and concurrent reduction of TOC in the microcosms (Fig. 4). Individual IRBs are capable of using a wide variety of organic substrates as an electron source for respiratory energy generation, including short-chain fatty acids (Fredrickson et al. 2008; Lovley et al. 1992), low molecular weight petroleum organics (Jahn et al. 2005; Kazumi et al. 1995; Lovley et al. 1989; Lovley and Lonergan 1990; Lu et al. 2008), aromatic compounds (Lovley et al. 1989; Lovley and Lonergan 1990; Lovley et al. 1992; Lu et al. 2008), and even extracellular DNA (Gödeke et al. 2011). With the assistance of other members in the complex MIR community, some of which may be equipped to degrade other bacterial cellular components (Mitchell and Nevo 1965), the MIR community could efficiently degrade the inactivated *E. coli* cells and couple that to dissimilatory iron reduction. This coupling potentially provides an ecological impetus for the MIR community to inactivate exogenous *E. coli* cells, providing a positive feedback loop as more Fe^2+^ inactivates more *E. coli* cells, which leads to higher MIR activity and higher Fe^2+^.

Since MIR is an important biogeochemical process in sedimentary and subsurface environments and is known to enhance the biodegradation of a large variety of organic pollutants, MIR is expected to influence water quality in such environments (Lovley 2006). Results from this study, for the first time, demonstrated that the MIR activity can also significantly enhance the inactivation of *E. coli*, and by inference other fecal bacteria. The production of Fe^2+^ as the metabolic waste of MIR was identified as a mechanism in *E. coli* inactivation under the anaerobic condition. Since the inactivated *E. coli* cells were shown to be used by the MIR community as an electron source to drive Fe(III) reduction, this represents a new mechanism for bacterial interspecies competition. This knowledge could further improve our understanding of the fate of fecal bacteria in natural sedimentary and subsurface environments where the MIR process is prevalent, and may also be explored for the enhancement of pathogen removal in many engineering processes, such as stormwater bioretention facilities, aquifer artificial recharge, and low-cost soil-based water reclamation.

## Electronic supplementary material

ESM 1 (DOC 205 kb)

## Data Availability

Not applicable.
