# Supplementary material for: Inactivation of Escherichia coli enhanced by anaerobic microbial iron reduction
Source: Environ Sci Pollut Res Int. 2020 Oct 20;28(45):63614–22. doi: 10.1007/s11356-020-11209-w (PMC8610951; doi:10.1007/s11356-020-11209-w)
Supplement: Supplementary file 1 — (DOC 205 kb) [file 11356_2020_11209_MOESM1_ESM.doc]

**Table S1**. Phylogenetic affiliation of OTUs with a relative abundance (RA) larger than 5% in the pooled anaerobic innocula, and identification of the iron reducing bacteria, denitrifer, sulfate reducing bacteria, and methanogens.

| OTUs | Taxon ^a^ | RA (%) | Putative function | Ref. |
| --- | --- | --- | --- | --- |
| 1 | *Erysipelotrichaceae* (f) | 20.9 |  |  |
| 2 | *Fusibacter* | 9.3 |  |  |
| 3 | *Geobacter* | 5.7 | IRB |  |
| 4 | *Bacteroidales* (o) | 5.2 |  |  |
| 5 | *Comamonadaceae* (f) | 4.1 |  |  |
| 6 | *Azospira* | 3.8 | Denitrifier |  |
| 7 | *Azoarcus* | 3.7 |  |  |
| 8 | *Pelobacteraceae* (f) | 2.8 |  |  |
| 9 | *Betaproteobacteria* (c) | 2.5 |  |  |
| 10 | *Bacteria* (k) | 2.5 |  |  |
| 11 | *Desulfobulbaceae* (f) | 2.3 | SRB |  |
| 12 | *Bacteroidales* (o) | 1.9 |  |  |
| 13 | *Thauera* | 1.8 |  |  |
| 14 | *Dechloromonas* | 1.8 |  |  |
| 15 | *Treponema* | 1.6 |  |  |
| 16 | *Bacteroidetes* (p) | 1.5 |  |  |
| 17 | *Desulfuromonadales* (o) | 1.2 |  |  |
| 18 | *Acholeplasma* | 1.0 |  |  |
| 19 | *Spirochaetes* (p) | 1.0 |  |  |
| 20 | *Candidatus Solibacter* | 0.9 |  |  |
| 21 | *Rhodocyclaceae* (f) | 0.9 |  |  |
| 22 | *Methanosarcina* | 0.9 | Methanogen |  |
| 23 | *Syntrophobacter* | 0.8 |  |  |
| 24 | *Proteobacteria* (p) | 0.8 |  |  |
| 25 | *Ruminococcaceae* (f) | 0.7 |  |  |
| 26 | *Clostridiaceae* (f) | 0.6 |  |  |
| 27 | *Cyclobacteriaceae* (f) | 0.6 |  |  |
| 28 | *Pedosphaerales* (o) | 0.5 |  |  |

^a^ The taxon of the OTUs were by default resolved at the genus level. The OTUs taxon followed by (k), (p), (c), (o), and (f) only found matches in the database at kingdom, phylum, class, order, and family levels, respectively.


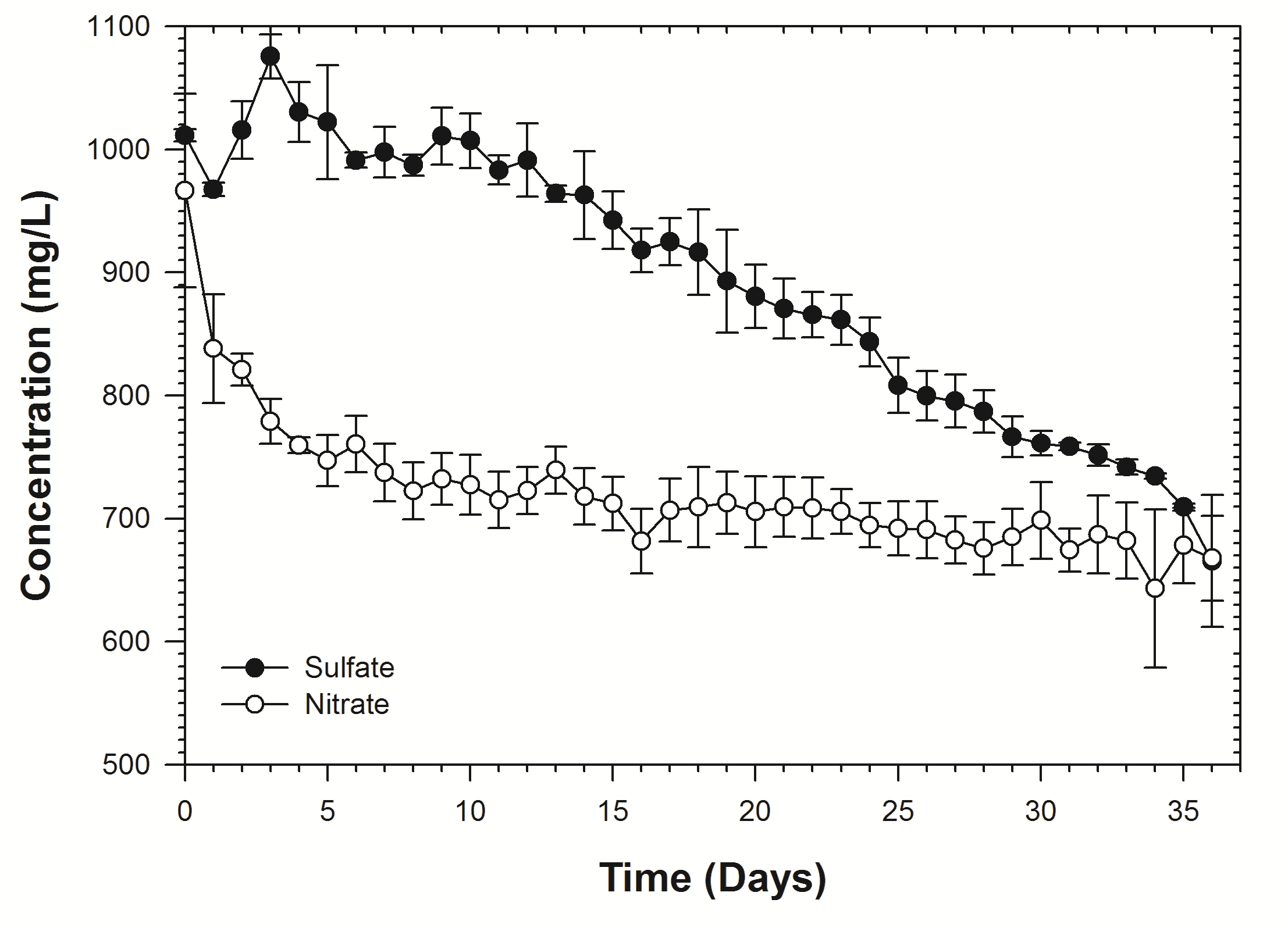


**Figure S1**. Reduction of sulfate and nitrate in the microbial sulfate reduction and microbial nitrate reduction microcosms over time. The error bars indicate the standard deviation of the mean of triplicate microcosms.

**References**

1. Methe, B. A.; Nelson, K. E.; Eisen, J. A.; Paulsen, I. T.; Nelson, W.; Heidelberg, J. F.; Wu, D.; Wu, M.; Ward, N.; Beanan, M. J.; Dodson, R. J.; Madupu, R.; Brinkac, L. M.; Daugherty, S. C.; DeBoy, R. T.; Durkin, A. S.; Gwinn, M.; Kolonay, J. F.; Sullivan, S. A.; Haft, D. H.; Selengut, J.; Davidsen, T. M.; Zafar, N.; White, O.; Tran, B.; Romero, C.; Forberger, H. A.; Weidman, J.; Khouri, H.; Feldblyum, T. V.; Utterback, T. R.; Van Aken, S. E.; Lovley, D. R.; Fraser, C. M., Genome of Geobacter sulfurreducens: metal reduction in subsurface environments. *Science* **2003,** *302*, (5652), 1967-9.

2. Hutchison, J. M.; Poust, S. K.; Kumar, M.; Cropek, D. M.; MacAllister, I. E.; Arnett, C. M.; Zilles, J. L., Perchlorate Reduction Using Free and Encapsulated Azospira oryzae Enzymes. *Environ Sci Technol* **2013,** *47*, (17), 9934-9941.

3. Pfeffer, C.; Larsen, S.; Song, J.; Dong, M. D.; Besenbacher, F.; Meyer, R. L.; Kjeldsen, K. U.; Schreiber, L.; Gorby, Y. A.; El-Naggar, M. Y.; Leung, K. M.; Schramm, A.; Risgaard-Petersen, N.; Nielsen, L. P., Filamentous bacteria transport electrons over centimetre distances. *Nature* **2012,** *491*, (7423), 218-221.

4. Smith, M. R.; Mah, R. A., Growth and methanogenesis by Methanosarcina strain 227 on acetate and methanol. *Applied and environmental microbiology* **1978,** *36*, (6), 870-9.
